# PD37 - Allergic disease may confer some protection against acute limphoblastic leukemia?

**DOI:** 10.1186/2045-7022-4-S1-P37

**Published:** 2014-02-28

**Authors:** Conceicao Nune Joacilda, Jose Angelo Rizzo, Decio Medeiros, Almerinda Rego Silva, Emanuel Sarinho

**Affiliations:** 1 Universidade Federal de Pernambuco, Recife, Brazil

## Background

Acute lymphoid leukemia is the most common pediatric cancer. Several factors may be found associated with leukemic state and previous allergic disease needs to be evaluated.

## Objective

To investigate a possible association between preceding history of allergy disease and high Total IgE levels with Acute Lymphoid Leukemia in children and adolescents.

## Methods

A hospital-based case-control study in the northeast region of Brazil. The population included 60 patients diagnosed with non-T acute lymphoid leukemia by means of bone marrow myelogram and immunophenotyping and 120 controls selected proportionally for age and gender. Data collection employed a structured questionnaire to determine prior history of allergies, as well as total IgE serum tests and clinical evaluations.

## Results

The results show that both total IgE serum levels and preceding history of asthma have a significant and inverse risk association with acute lymphoid leukemia.

In the unadjusted model, the exposure variables with a p-value < 0.20 were: asthma, high IgE levels, concomitant allergic diseases, atopic dermatitis, allergic rhinitis and urticaria. The inverse relation to both asthma and IgE serum levels continued to be significant in the adjusted model, showing significant p-value 0.044 and OR (CI 95%) of 0.14 (0.02 – 0.95); and p-value 0.001 and OR (CI 95%) of 0.10 (0.02 – 0.41), respectively.

## Conclusion

High levels of total IgE, especially related to asthma, appear to contribute to alterations in the immune system, which may activate Th2-mediated pathways and this fact might confer some protective role against acute lymphoid leukemia.

